# Nutritional Potential of Selected Insect Species Reared on the Island of Sumatra

**DOI:** 10.3390/ijerph14050521

**Published:** 2017-05-12

**Authors:** Anna Adámková, Jiří Mlček, Lenka Kouřimská, Marie Borkovcová, Tomáš Bušina, Martin Adámek, Martina Bednářová, Jan Krajsa

**Affiliations:** 1 Department of Quality of Agricultural Products, Faculty of Agrobiology, Food and Natural Resources, Czech University of Life Sciences Prague, 165 21 Prague, Czech Republic; adamkovaa@af.czu.cz; 2 Department of Food Analysis and Chemistry, Tomas Bata University in Zlin, 760 01 Zlin, Czech Republic; 3 Department of Microbiology, Nutrition and Dietetics, Faculty of Agrobiology, Food and Natural Resources, Czech University of Life Sciences Prague, 165 21 Prague, Czech Republic; kourimska@af.czu.cz; 4 Department of Zoology, Fisheries, Hydrobiology and Apiculture, Mendel University, 613 00 Brno, Czech Republic; borkov@mendelu.cz; 5 Department of Husbandry and Ethology of Animals, Faculty of Agrobiology, Food and Natural Resources, Czech University of Life Sciences Prague, 165 21 Prague, Czech Republic; businat@af.czu.cz; 6 Department of Microelectronics, Faculty of Electrical Engineering and Communication, Brno University of Technology, 616 00 Brno, Czech Republic; adamek@feec.vutbr.cz; 7 Department of Information Technology, Mendel University, 613 00 Brno, Czech Republic; bednarova@mendelu.cz; 8 Department of Forensic Medicine, Faculty of Medicine, Masaryk University, 601 77 Brno, Czech Republic; jan.krajsa@mail.muni.cz

**Keywords:** edible insect, *Tenebrio molitor*, *Zophobas morio*, *Gryllus assimilis*, crude protein, fats, amino acid profile, chitin, Indonesia

## Abstract

Inhabitants of the Indonesian island of Sumatra are faced with the problem of insufficient food supplies and the consequent risk of undernourishment and health issues. Edible insects as a traditional and readily available food source could be part of the solution. The nutritional value of insects depends on many factors, e.g., species, developmental stage, sex, diet, and climatic conditions. However, edible insects bred in Sumatra for human consumption have never before been assessed with regard to their nutritional value. Our study involved analyses of crude protein, chitin, fat and selected fatty acid contents of giant mealworm larvae (*Zophobas morio*), larvae of the common mealworm (*Tenebrio molitor)* and nymphs of the field cricket (*Gryllus assimilis*). Crude protein content in the samples ranged from 46% to 56%. Highest (35%) and lowest (31%) amounts of fat were recorded in giant mealworm larvae and larvae of the common mealworm, respectively. Chitin amounts ranged from 6% to 13%. Based on these values, which are comparable to those known from other food insects reared in different regions of the world, the edible species bred in Sumatra could become food sources with a potential to help stave off hunger and undernourishment.

## 1. Introduction

Throughout Indonesia, 20 million people suffer from undernourishment, which is approximately 8% of the Indonesian population [1]. In Indonesia, one in every five children suffers from malnutrition and one in every three children suffers from from stunting [2]. In the tropical Indonesian island of Sumatra, the diet consists mainly of rice, fruit and vegetables; meat, dairy products and bread are minor components [3].

Edible insects are one of the traditional, readily available and nutritious foods [4,5] in Indonesia, and can be a source of animal protein and fat. However, insects are consumed randomly, and not systematically implemented in the menu. Knowledge of the nutritional values of edible insect in this area is not sufficient. This creates a blank field in the evaluation of the menu of the indigenous inhabitants. Therefore, the subsequent influence of edible insect consumption on the health of the local people is not yet known [6].

Thanks to its nutritional composition, edible insects can be a good food source for the local population, and can, to a certain extent, alleviate the problem of malnutrition [7].

The aim of this work is to analyze the nutritional values of selected edible insect species, reared on the island of Sumatra, as a traditional and readily available food source for the indigenous inhabitants. This aim was chosen due to the missing complex data about nutritional values of edible insects reared in this area. Results are compared with nutritional analyses of edible insects from different regions of the world.

For easy breeding (successfully mastered at an industrial level in the following three species), *Tenebrio molitor, Gryllus assimilis* and *Zophobas morio* are chosen and used for our analyses. They are, to varying degrees, able to dispose of waste grain and vegetable and fruit scraps from markets [8,9]. Apart from the feed, the nutritional value of edible insects is influenced mainly by other factors—such as species, developmental stage, sex, diet and the environment [10,11,12]. As stated by Reference [13], the species itself is not as important as the composition of the insect feed.

## 2. Materials and Methods

### 2.1. Materials

Samples used for analyses were: larvae of the giant mealworm beetle (*Zophobas morio*), larvae of the common mealworm beetle (*Tenebrio molitor*, common mealworm larvae—CML) and nymphs of the field cricket (*Gryllus assimilis*). Samples were imported from the island of Sumatra, where the insects were bred in the local insect breeding facility at the optimum conditions for the development of each species. Insects were fed with a mixture of organic waste from the local marketplace—vegetable parts (carrots, cabbage, Chinese cabbage, tomatoes, potatoes), fruits (banana, orange), mustard seed, and chicken. Insect samples were processed as follows: larvae in the last and penultimate instar development (with the full length of the body just before pupation, penultimate and last instar) were collected. The next step was starving for 48 h, killing with boiling water (100 °C, 2 min) and drying by gas oven (100 °C) to a constant weight under conditions allowed by the field drying in Sumatra. The prepared samples were homogenized, stored in a plastic box and transported to the laboratory where they were stored in a refrigerator box at 4–7 °C until analysis (four weeks). Each sample was analyzed three times.

### 2.2. Methods

#### 2.2.1. Nitrogen and the Crude Protein Content Determination by Kjeldahl

Nitrogen and crude proteins were analyzed using the Kjeldahl method [14]. The samples (1 g) were mineralized at 420 °C for 105 min. Distillation was performed on a Kjeltec™ 2200 (FOSS, Hillerød, Denmark) for 4 min. The content of crude protein was calculated by multiplying the N content by the coefficient 6.25.

The calculation of crude protein (%) was performed according to the following formula:

CP% = (sample N content − N content in chitin) × 6.25
(1)

#### 2.2.2. Determination of Fat Content by Soxhlet

The determination of fat content was performed by extraction using the Soxhlet method [15] with Gerhardt Soxtherm SOX414 (C. Gerhardt GmbH & Co. KG, Königswinter, Germany). 5 g of dried and homogenized sample (accurate to 0.0001 g), were placed in an extraction thimble and extracted with 150 mL of petroleum ether (program: 70 °C for 120 min). The extracted sample was then dried at 103 °C and weighed until a constant sample weight was achieved.

#### 2.2.3. Determination of Chitin

Chitin in the samples was determined by hydrolysis by Liu et al. [16]. The weight of the samples was 4 × 1.6 g (2× for the determination of chitin and 2× for the determination of nitrogen in chitin). Then, the sample was hydrolyzed for 30 min in 100 mL of 1 M HCl at 100 °C. The resulting hydrolysate was filtered and washed with 500 mL of hot distilled water until a neutral pH was achieved. Because insects contain a relatively large amount of fat, which slowed or completely stopped the filtration, a small amount of 5% KOH was added during the filtration, which caused the saponification of fats, thereby increasing their solubility in water and enabling the washing the sample with the required amount of distilled water. To each filtered sample, 100 mL of 1 M NaOH was added and the samples were again hydrolyzed for 20 h at 80 °C. The hydrolysate was then filtered again using fritted glass for fibre and washed with 500 mL of hot water to a neutral pH. Two filtered hydrolysates from each sample were dried, weighed, incinerated and weighed again, and then the chitin content was calculated using the description below. The other two samples were transferred to mineralization flasks, and the nitrogen contained in the chitin was determined by the Kjeldahl method.

The calculation of chitin (%) was performed according to the following formula:

Chitin% = ((weight of the dry matter − weight of burnt fritted glass)/sample weight) × 100
(2)

#### 2.2.4. Determination of the Fatty Acid Profile

The esterification of lipids was carried out using Soxhlet extraction in accordance with ISO 12966-2:2011 [17], using a standard 0.25 N methanolic KOH. Fatty acid methyl esters were analyzed using a modified method of AOAC 996.06 on the gas chromatograph system Agilent 7890 GC (Agilent Technologies, Santa Clara, CA, USA) with flame ionization detector (detector temperature: 250 °C) equipped with a Restek column Rt^®^-2560 (100 × 0.25 mm ID × 0.2 µm film) from Restek Corporation (Bellefonte, PA, USA). Hexane was used as solvent and a sample volume of 1 μL was injected in the split mode (ratio 20:1) to the injector heated at 225 °C. The initial oven temperature was 70 °C (holding time 2 min), Ramp1 to 225 °C at 5 °C/min (holding time 9 min), Ramp2 to 240 °C at 5 °C/min (15 min long). Helium was used as the carrier gas with a flow rate of 1.2 mL/min. The results of fatty acid profiles were identified through the standard Food Industry FAME Mix, cat.# 35077, from Restek Corporation (Bellefonte, PA, USA).

#### 2.2.5. Statistical Analysis

The data were processed using Excel 2013 (Microsoft Corporation, Redmond, WA, USA) and STATISTICA Cz version 12 (StatSoft, Inc., Tulsa, OK, USA). Results were expressed by an average ± standard deviation. A comparison of each species concerning the content of crude protein, lipid, and chitin using the analysis of variance of mean values (Parametric ANOVA) was not carried out due to the small sample size and the failure to observe basic assumptions for this analysis. The comparison was evaluated using non-parametric multiple comparison of *p*-values after the Kruskal-Wallis test and the median test with a significance level of *p* = 0.05.

## 3. Results

The results of crude protein content for each species of edible insects in dry matter are shown in Table 1. Measured data showed a statistically significant difference (*p* < 0.05) between the field cricket (*Gryllus assimilis*) and giant mealworm (*Zophobas morio*). For other species, the difference between species was not confirmed.

Further, the measurement of fat content was carried out using Soxhlet’s method; the results are shown in Table 2. For the fat content in the selected species, the difference between species was not confirmed.

Furthermore, the determination of chitin using hydrolyzing with HCl and NaOH was carried out. The results are shown in Table 3. For chitin content, a statistically significant difference (*p* < 0.05) between common mealworm (*Tenebrio molitor*) and other species was confirmed. For other species, the difference between species was not confirmed.

The results of selected fatty acids content gained during the research were subsequently compared with other studies as the percentage of each selected fatty acid in the total amount of selected fatty acids, as shown in Table 4, Table 5 and Table 6. The proportion of oleic acid was found to be predominant in the oil of giant mealworm larvae, CML, and pupae, while linoleic acid was the most abundant in the oil of field cricket nymph.

## 4. Discussion

As early as 1975, Reference [7] pointed out that the one way to help solve the problem of undernourishment in the world would be the use of edible insects, which are readily available and nutritious [4,5]. Edible insects have high protein content, which has exceptional importance in a healthy diet and cannot be replaced by other nutrients. Also, the content of all essential amino acids, which have to be received through food, is high [22]. Proteins are of high quality [24] and, besides being eaten, they can be used in other food-related branches [25]. Another protein quality indicator is its digestibility, the usability by the human body. According to Reference [19], insect protein digestibility is as high as 86–89%.

Crude protein content in insects is generally within the range of 40 to 75 g/100 g in dry matter, which is comparable to the crude protein content in common commodities of animal origin [26]. Reference [27] declared a greater range of 15% to 81%. The crude protein content in CML (*Tenebrio molitor*) was in accordance with the literature. Reference [18] stated the content in this species to be 50.9%; Reference [28] reported 49.1%; and Reference [29] stated the value of 45.1–48.6%, depending on the feed. Similarly, in the giant mealworm beetle (*Zophobas morio*), our result corresponds with Reference [20]—46.8%. Reference [18] stated the content to be 54.3% in the same species, and Reference [29] stated the content to be between 34.2–42.5%, depending on the feed. In the field cricket (*Gryllus assimilis*), the crude protein content from our research is similar to Reference [18], who detected the value of 59.2%. Reference [28] stated a content of 46.8% in a similar species—home cricket (*Acheta domesticus*). Analyses confirm Reference [29], that differences up to 11% can be caused by a different feed composition.

Edible insects display great variability considerng not only proteins, but also fats. Fat is an energy source, and its content may be from 7 to 77 g/100 g of edible insect dry matter [30]. These variabilities depend on the season, development stage, sex, environment and feed. Fat content was detected within the range of 31% to 35%. In CML, the value was 31%. This value is in accordance with the references, that state, e.g., 32.0% [31], 36.1% [18], 35.0% [28], 27.1% (recalculated from the content in fresh weight 9.9% [24]) or 18.9–38.3% depending on the feed composition [29]. This value is the same as stated by Reference [32], who found a fat content of 34.54% in *Tenebrio molitor* larvae. Fat content in larvae did not differ significantly from that of pupae (32%). The highest content of fat among the analyzed samples was the giant mealworm (larva) with 35%, which could be a suitable source of nutritional energy. Reference [29] stated the content to be between 32.8% and 43.5% in the same species, depending on the feed, while Reference [28] reported 42.0% and Reference [18] declared the value of 40.3%. Similarly, the measured value of 32% for field crickets is in accordance with the literature. Reference [18] stated that the value is 34.3%. Reference [28] declared in a similar species, the house cricket, to be only 14.4%, similar to Reference [24], who declared the value of 12.3% (recalculated from the content in fresh weight 3.6%) or Reference [31], which reported 15.3%. The majority of the most common species of edible insects is thus comparable with some traditional foods such as eel meat (30% dry matter), pork (32% dry matter) or young goose meat (36% dry matter) [33,34].

Analyses of the fatty acid profile were aimed at the essential fatty acid profile, especially the content of linoleic acid, which is important for physiological processes and the creation of linolenic acid. Another polyunsaturated fatty acid (PUFA) benefit is the prevention of cardiovascular diseases [22]. Polyenic fatty acids in edible insect fatty acid profiles may comprise up to 70% of the total fats [35]. According to the analyses of Reference [36], the minimal total content of polyenic fatty acids in termites was 5.9% to 12.2% of the total fat. Reference [36] found that the most abundant fatty acids were C18, among them the oleic acid C18:1, the content of which was from 41.7% to 50.2% of the total fat in termites. In the case of the larvae of *Zophobas morio* and *Tenebrio molitor*, the content presented is the same or lower [19,20,21]. Also, linoleic acid C18:2, linolenic acid C18:3 and saturated stearic acid C18:0 belong to C18 fatty acids [36]. The ratio of n-6 and n-3 fatty acids is mostly 5.8:10 to 57.7:10 [37]. From a nutritional point of view, they are important for the proper development of the brain and nervous system in children and newborns. In developing countries, insect fat could cover the deficit of n-3 and n-6 fatty acids [38]. Also, saturated fatty acids (palmitic acid, stearic acid) have a significant content in the fatty acid profile.

A comparison of the fatty acid profile of giant mealworm larvae (*Zophobas morio*) showed that the sequence of the first four fatty acids (C18:1 (cis-9), C18:2 (cis-9,12), C16:0, C18:0) is identical to reports by other authors, as well as in CML (*Tenebrio molitor*). Only Reference [21] documented myristic acid in the fourth place, unlike other authors, who report stearic acid as the fourth. In the field cricket (*Gryllus assimilis*) and related species house cricket (*Acheta domesticus*), many studies showed linoleic acid as the most abundant, as was the case in our analyzed samples. Reference [39] detected alpha-linolenic acid as the most abundant in the samples of *Chorthippus parallelus*, representing up to 40.4% of the total fatty acid content. On the contrary, Reference [21] declared oleic acid as the most plentiful in the samples of (*Acheta domesticus*), while other papers placed it second or third (e.g., [19,20]), as was the case in our analysed samples. Oleic and palmoitic acids are equally abundant. Stearic acid is in the fourth place in our analyses and also in the literature.

According to Reference [40], the content of fatty acids is determined by several factors such as species, life-cycle phase, environmental factors and nutrition. A comparison of the fatty acid profile with species bred in Czech Republic with controlled feed, analyzed by Reference [41], proved differences dependening on the breeding location. The comparison also revealed differences in essential fatty acid proportions (linoleic and linolenic acid), as the samples from Sumatra had higher content of these fatty acids.

Chitin is considered as an indigestible fibre with protective effects on human health [42,43], even though the enzyme chitinase is found in human gastric juices [44]. However, it was found that this enzyme may be inactive. In this case, only in saliva and in the stomach is it partially hydrolyzed by lysozyme and hydrochloric acid. Active chitinase response in the body prevails among people from tropical countries where the consumption of insects has a long-term tradition [38]. Chitin is present in the cuticles of arthropods, which can be hardened (e.g., by crabs, crayfish, insects) and transformed into exoskeleton [45]. The removal of chitin improves the digestibility of insect protein [46].

The composition and amount of chitin in insects varies according to the species and development stage. For the common mealworm, Reference [47] gave an average chitin content of 5%; Reference [48] reported 1.2%, and Reference [49] declared the average chitin content of insects to be 10%. In *Cirina forda* larvae, the chitin content is 9.4% [50]. In field cricket (*Gryllus testaceus walker*), Reference [43] declared 8.7% of chitin in dry matter. This is in accordance with our value of 7%. Our result of chitin content in the larval stage of the same species was 12%. Chitin content is influenced by amino acids [51]. Chitin content in giant mealworm larvae in our research was 6% in dry matter. The available literature does not mention the chitin content in this species.

## 5. Conclusions

This research aimed to determine the nutritional composition (crude protein content, lipids and chitin) in three species of edible insects: common mealworm, giant mealworm and field cricket reared on the island of Sumatra. Essential fatty acids, that are presumed to reduce the incidence of cardiovascular diseases, were detected in the samples. In CML, common mealworm pupae and giant mealworm larvae; oleic acid was found to predominate the oil while linoleic acid was the most abundant in the oil of field cricket nymph. Our study also proved a significant content of chitin, which has a protective impact on human health, in the evaluated species of edible insects. The obtained nutritional values of the analyzed samples of edible insects were further compared with other commodities of animal origin. Edible insects are beneficial from a nutritional point of view, because they can serve as substitutes in the Indonesian region of Sumatra for other foods of animal origin, which are insufficient in the local diet. This study confirms that edible insects in Sumatra can be a strategic solution for hunger problems and subsequent undernourishment.

## Figures and Tables

**Table 1:** Crude protein content for each species of edible insects in dry matter.

Species	Crude Protein [g/100 g]
Giant mealworm larva	46 ± 1.0 ^a^
Common mealworm pupa	51 ± 1.0 ^b^
Common mealworm larvae	52 ± 0.4 ^b^
Field cricket nymph	56 ± 3.1 ^b^

^a,b^ The different superscripts express belonging to the statistically significantly different groups, *p* < 0.05.

**Table 2:** The fat content for each species of edible insects.

Species	Fat [g/100 g]
Giant mealworm larva	35 ± 0.1 ^a^
Common mealworm pupa	32 ± 0.5 ^a^
Common mealworm larvae	31 ± 1.1 ^a^
Field cricket nymph	32 ± 0.2 ^a^

^a^ The different superscripts express belonging to the statistically significantly different groups, *p* < 0.05.

**Table 3:** The content of chitin for each species of edible insects.

Species	Chitin [g/100 g]
Giant mealworm larva	6 ± 0.8 ^a^
Common mealworm pupa	12 ± 0.2 ^b^
Common mealworm larvae	13 ± 0.4 ^b^
Field cricket nymph	7 ± 0.7 ^a^

^a,b^ The different superscripts express belonging to the statistically significantly different groups, *p* < 0.05.

**Table 4:** Selected fatty acid content in giant mealworm larvae (*Zophobas morio*—ZM) (% of total fatty acids).

Origin	Sumatra	Brno	Marion	Spain
Stage	Larvae	Larvae	Larvae	Larvae
C12:0	0.7 ± 0.1	0.0	-	0.0
C14:0	1.4 ± 0.1	0.6	1.0	1.1
C16:0	29.1 ± 0.6	32.2	31.3	30.6
C16:1 (cis-9)	1.2 ± 0.1	1.2	0.4	1.0
C17:0	0.2 ± 0.1	0.1	0.4	0.0
C18:0	6.4 ± 0.3	7.7	7.5	7.7
C18:1 (cis-9)	35.7 ± 0.3	35.9	39.1	35.2
C18:2 (cis-9,12)	23.4 ± 0.3	19.8	19.5	22.9
C20:0	0.1 ± 0.1	2.4	0.2	-
C18:3 (cis-9,12,15)	1.6 ± 0.1	0.0	0.7	1.4
Reference		Bednářová, 2013, [18]	Finke, 2002, [19]	Barroso, 2014, [20]

**Table 5:** Selected fatty acid content in common mealworm larvae (*Tenebrio molitor*) (% of total fatty acids).

Origin	Sumatra	Sumatra	The Netherlands	Warsaw	Marion	Spain	Spain	Marion
Stage	Pupa	Larva	-	-	Larva	Larva	Larva	Adult
C12:0	0.2 ± 0.1	0.3 ± 0.1	0.2	0.2	-	-	0.0	-
C14:0	2.5 ± 0.2	2.6 ± 0.1	3.2	2.6	2.3	2.2	2.2	1.8
C16:0	21.3 ± 0.1	20.2 ± 0.3	18.8	18.1	18.5	16.7	16.7	18.9
C16:1 (cis-9)	0.2 ± 0.1	0.4 ± 0.1	2.1	2.1	2.8	2.2	2.2	1.3
C17:0	0.2 ± 0.1	0.3 ± 0.1	0.0	0.2	-	-	0.0	0.4
C18:0	4.8 ± 0.2	4.3 ± 0.5	2.5	3.9	3.2	3.4	3.4	5.8
C18:1 (cis-9)	36.3 ± 0.3	37.7 ± 0.6	50.2	41.2	43.6	43.8	43.8	39.9
C18:2 (cis-9,12)	31.9 ± 0.5	31.9 ± 0.2	22.1	29.9	28.2	30.5	30.6	30.5
C20:0	0.7 ± 0.1	0.6 ± 0.1	0.0	0.2	0.2	-	-	0.4
C18:3 (cis-9,12,15)	1.8 ± 0.1	1.7 ± 0.1	0.9	1.6	1.1	1.1	1.1	0.9
Reference			Tzompa-Sosa, 2014, [21]	Zielińska, 2015, [22]	Finke, 2002, [19]	Sánches-Muros, 2016, [23]	Barroso, 2014, [20]	Finke, 2002, [19]

**Table 6:** Selected fatty acid content in field cricket (*Gryllus assimilis*—GA) and house cricket (*Acheta domesticus*—AD) (% of total fatty acids).

Origin	Sumatra	Spain	Marion	Marion	The Netherlands	Spain
Kind	GA	GA	AD	AD	AD	AD
Stage	Nymph	Adult	Adult	Nymph	-	Adult
C12:0	2.7 ± 0.1	0.0	-	-	0.3	0.0
C14:0	0.7 ± 0.2	0.4	0.6	0.7	1.9	0.5
C16:0	22.0 ± 0.5	27.0	25.1	22.0	27.0	25.2
C16:1 (cis-9)	1.3 ± 0.1	1.7	1.4	1.1	2.2	0.9
C17:0	1.2 ± 0.1	0.0	0.3	0.4	0.2	0.0
C18:0	8.2 ± 0.3	7.4	9.3	10.5	6.3	8.9
C18:1 (cis-9)	25.5 ± 0.5	26.4	24.8	23.1	30.3	20.7
C18:2 (cis-9,12)	35.7 ± 0.3	35.2	36.8	39.7	30.2	41.9
C20:0	1.3 ± 0.1	-	0.6	1.1	0.0	-
C18:3 (cis-9,12,15)	1.3 ± 0.1	1.8	1.0	1.4	1.6	1.8
Reference		Barroso, 2014, [20]	Finke, 2002, [19]	Finke, 2002, [19]	Tzompa-Sosa, 2014, [21]	Barroso, 2014, [20]
